# Impact of immunosuppressive agents on the expression of indoleamine 2,3-dioxygenase, heme oxygenase-1 and interleukin-7 in mesangial cells

**DOI:** 10.3892/mmr.2015.3713

**Published:** 2015-05-04

**Authors:** GUO-BIAO LIANG, GUANG-HENG LUO, DING-SU BAO, AN-JIAN CHEN, YONG-XIANG ZHUANG, YA-NAN GUO, XIN WANG, YUAN-LIANG WANG, ZONG-PING CHEN, YI-PING LU, YOU-PING LI

**Affiliations:** 1 Department of Urology, Affiliated Hospital of Zunyi Medical College, Zunyi, Guizhou 563003, P.R. China; 2 Department of Urology, Guizhou Provincial People’s Hospital, Guiyang, Guizhou 550005, P.R. China; 3 Department of Urology, West China Hospital, Sichuan University, Chengdu, Sichuan 610041, P.R. China; 4 Transplantation Immunology Laboratory, West China Hospital, Sichuan University, Chengdu, Sichuan 610041, P.R. China

**Keywords:** indoleamine 2, 3-dioxygenase, heme oxygenase-1, interleukin-7, mesangial cell, immunosuppressive agents, *in vitro*

## Abstract

Chronic allograft nephropathy (CAN) is a major cause of graft loss following kidney transplantation and may result from the interactions of various immune and non-immune factors. The aim of the present study was to establish an *in vitro* model of glomerular mesangial cell injury in order to examine the gene expression levels of indoleamine 2,3-dioxygenase (IDO), heme oxygenase-1 (HO-1) and interleukin-7 (IL-7) in mesangial cells during the healing process as well as to investigate the effects of various immunosuppressants on the expression of these genes. The HBZY-1 glomerular mesangial cell line was pre-treated *in vitro* with cytochalasin B for 2 h to induce reversible damage. Following the pre-treatment, the HBZY-1 cells were divided into five groups: Blank control group, cyclosporine A (CsA) group, tacrolimus (Tac) group, mycophenolate mofetil (MMF) group and rapamycin (RAPA) group. After treating the mesangial cells with each immunosuppressive drug for 6, 12 or 24 h, the mRNA and protein expression levels of IDO, HO-1 and IL-7 were examined using reverse transcription quantitative polymerase chain reaction (RT-qPCR), western blot and immunohistochemical analyses. The results showed that expression levels of HO-1 were significantly upregulated in response to treatment with CsA, FK506, RAPA and MMF, whereas the expression levels of IL-7 were markedly downregulated by treatment with the above immunosuppressants. CsA, FK506 and MMF significantly enhanced the expression levels of IDO, whereas RAPA exhibited no apparent effect on IDO. The present study may contribute to the understanding of the pathogenesis of CAN and provide novel strategies for the prevention and treatment of CAN.

## Introduction

Chronic allograft nephropathy (CAN) is a major cause of graft loss following kidney transplantation (1). CAN is characterized by renal interstitial fibrosis and tubular atrophy and may result from the interactions of various immune and non-immune factors (2). The glomerular mesangium has an important role in the development of inflammatory diseases, and it has previously been demonstrated that strategies that suppress the adverse proliferation of mesangial cells are able to attenuate inflammatory kidney diseases (3). Glomerular mesangial cells represent a specialized type of vascular pericyte and are the most active intrinsic renal cells in glomeruli (4,5). Mesangial cell proliferation or dysfunction results in the excessive deposition of mesangial matrix, which subsequently leads to glomerular mesangial sclerosis and renal interstitial fibrosis (6–8). Indoleamine 2,3-dioxygenase (IDO) is a 45 kD iron-containing heme enzyme monomer, which is the rate-limiting enzyme for the metabolism of tryptophan, particularly in dendrites. In addition, IDO is an effective immunosuppressive enzyme (9–11). Tryptophan is required for T cell proliferation and is an essential amino acid. IDO can be catalyzed by tryptophan degradation of serotonin anthranilic acid and 3-alanine, and can also inhibit T cell proliferation, accelerate the apoptosis of T cells and cause cell cycle arrest. In addition, IDO can enhance the ability of local antioxidant defenses, reducing damage caused by allograft hypoxia, or ischemia caused by rejection, thereby promoting long-term allograft survival (12,13). Heme oxygenase (HO) is an antioxidant enzyme, which is the rate-limiting enzyme of heme metabolism. HO degrades heme to biliverdin and carbon monoxide (14–20). HO includes three isoenzymes: HO-1, HO-2 and HO-3. HO-1 is also termed heat shock protein 32, which is expressed at low levels in the majority of tissues, with high-level expression being induced by stimulation injury (21–23). Expression of HO-1 has been shown to be induced in various organ transplantation models, where it has a role in protection, including the prevention of ischemia-reperfusion injury, in order to reduce the damage caused by acute, chronic and xenograft rejection (20). Due to the continued retention of memory CD8^+^ T cells, chronic rejection remains a major obstacle to achieving tolerance, and long-term memory T cells may pose a threat to allograft survival (10). Interleukin-7 (IL-7) is a T cell survival promoting cytokine, which maintains essential cellular homeostasis and regulates the expression of anti-apoptotic B-cell lymphoma-2 (21). Studies have demonstrated that IL-7 is important for the survival of memory T cells, especially for CD8^+^ memory T cell generation (22). Therefore, inhibition of the production of IL-7 may help suppress the generation of memory T cells, and prevent or delay the process of CAN, thus contributing to the long-term survival of allografts. The aim of the present study was to establish an *in vitro* model of glomerular mesangial cell injury in order to examine the gene expression levels of IDO, HO-1 and IL-7 in mesangial cells during the healing process. The present study also aimed to investigate the effects of various immunosuppressants on the expression of these genes. This study may contribute to the understanding of the pathogenesis of CAN and provide novel strategies for the prevention and treatment of CAN.

## Materials and methods

### Cells and reagents

The HBZY-1 glomerular mesangial cell line was obtained from the Laboratory of Transplant Engineering and Immunology, West China Hospital, Sichuan University (Chengdu, China). The Total RNA Isolation kit was purchased from Invitrogen Life Technologies (Carlsbad, CA, USA). The cDNA synthesis and polymerase chain reaction (PCR) kits were purchased from Thermo Fisher Scientific (Waltham, MA, USA). The primary and secondary antibodies used for western blot analysis for detecting the protein expression levels of IDO, HO-1 and IL-7 were purchased from Santa Cruz Biotechnology, Inc. (Austin, TX, USA) and Beijing Zhongshan Golden Bridge Biological Technology Co., Ltd. (Beijing, China), respectively. The immunohistochemistry detection kits used for analyzing the expression of IDO, HO-1 and IL-7 were purchased from Dako (Glostrup, Denmark) and Beijing Zhongshan Golden Bridge Biological Technology Co., Ltd. The same primary antibodies were used for western blotting and immunohistochemical analysis.

### Primer design and synthesis

The primers specific for each target gene were designed based on exon distribution and mRNA sequence, using the Primer Premier version 5.0 software (Premier Biosoft, Palo Alto, CA, USA). Each primer spanned >two exons and yielded products of 100–250 bp in length. The primers and TaqMan^®^ probes for IDO, HO-1, IL-7 and GAPDH were synthesized by Shenggong Biotech Co., Ltd. (Shanghai, China), and are presented in Table I. The housekeeping gene GAPDH was used as an internal reference.

### Cell culture and resuscitation

The HBZY-1 glomerular mesangial cell line was quickly thawed (<1 min) at 37°C. The cell suspension was then cultured overnight with fresh Dulbecco’s modified Eagle’s medium (DMEM; Hali Biological Engineering Co., Ltd., Chengdu, China) supplemented with 10% newborn calf serum (Sino-American Biotechnology Company, Luoyang, China) and penicillin/streptomycin (North China Pharmaceutical Corporation, Shijiazhuang, China), at 37°C in an incubator containing 5% CO_2_. The next day, the cells were washed twice with phosphate-buffered saline (PBS) (0.01 mol/l) and cultured in fresh medium. The medium was then discarded and the cells were washed twice with PBS (0.01 mol/l) and incubated with 0.25% trypsin solution (Sino-American Biotechnology Company) at 37°C for 2 min. Once the cells had become rounded, the trypsin digestion was terminated using fresh DMEM supplemented with 20% newborn calf serum. Cell suspensions were prepared and centrifuged at 1,300 x g for 5 min. Following removal of the supernatant, the cells were resuspended in fresh DMEM supplemented with 10% newborn calf serum and were cultured at 37°C in a humidified incubator containing 5% CO_2_.

### Experimental protocol

Cytochalasin B (Sigma West, San Francisco, CA, USA) was administered *in vitro* to introduce reversible damage to the glomerular mesangial cells. The effects of various immunosuppressants on the mRNA and protein expression of IDO, HO-1 and IL-7 in the mesangial cells during cellular repair were then determined. Briefly, the HBZY-1 proliferating mesangial cells were cultured *in vitro* and incubated with cytochalasin B (5 *µ*g/ml) for 2 h. Following pretreatment with cytochalasin B, the HBZY-1 cells were divided into the following five groups: Blank control group, in which the cells were treated with media only; cyclosporine A (CsA) group, in which the cells were incubated with 3 *µ*g/ml CsA (Sino-us East China Pharmaceutical Co., Ltd, Hangzhou, China); tacrolimus (Tac) group, in which the cells were incubated with 1 *µ*g/ml Tac (Fujisawa Ireland Ltd., Killorglin, Ireland); mycophenolate mofetil (MMF) group, in which the cells were treated with 0.3 *µ*g/ml MMF (Roche Pharmaceutical Co., Ltd, Shanghai, China); and rapamycin (RAPA) group, in which the cells were incubated with 10 ng/ml RAPA (Wyeth Pharmaceutical Co., Ltd., Philadelphia, PA, USA). The mRNA expression levels of IDO, HO-1 and IL-7 were analyzed at 6, 12 and 24 h after the administration of the drugs, using reverse transcription quantitative PCR (RT-qPCR). In addition, the protein expression levels of the three target proteins were examined by western blot analysis and immunohistochemistry.

### RT-qPCR analysis

Total RNA was extracted from the cultured cells (5×10^5^) using chloroform and further purified using the RNA Isolation kit. A portion of the total RNA was subjected to electrophoresis on a 1% agarose gel (5 *µ*l per lane) (Biowest Co., Ltd., Nuaillé, France). The remaining isolated total RNA was stored at −80°C until further use. cDNA was synthesized using the cDNA synthesis kit on a PCR machine and was then stored at −20°C. The primers for the amplification of IDO, HO-1, IL-7 and GAPDH, and the corresponding TaqMan^®^ probes, are listed in Table I. Quantitative analysis of the mRNA expression levels was conducted using a real-time PCR kit (Takara Biotechnology Co., Ltd., Dalian, China) on a Real-Time Quantitative Cycler (FTC2000; Funglyn Biotech, Inc., Ontario, Canada), according to the manufacturer’s instructions. The PCR cycling conditions were set as follows: Initial denaturation at 94°C for 2 min; then 45 cycles of denaturation at 94°C for 20 sec, refolding at 50/54°C for 20 sec and extension at 60°C for 30 sec then a final extension for 7 min. Amplification curves were created by plotting the fluorescence intensity of each sample against the cycle number, and were used to determine the amplification cycle number at which the fluorescence intensity of a sample exceeded a specific threshold, also known as the cycle threshold (Ct) value. GAPDH was used as an internal reference. PCR results were expressed as the ΔCt values (ΔCt=Ct_sample_ − Ct_GADPH_). The ΔCt values represent the relative abundance of the target gene. Larger ΔCt values indicate lower starting copy numbers of a gene and lower gene product abundance.

### Western blot analysis

The protein expression levels of IDO, HO-1 and IL-7 were determined in the mesangial cells 24 h after the administration of the drugs by western blot analysis. Preparation of cell lysates, protein fractionation and transfer were conducted according to product specifications. Following transfer to polyvinylidene difluoride membranes as described previously (24), 5% non-fat milk was used to block the membranes overnight. The membrane was then incubated with the following primary antibodies were used: Affinity purified rabbit polyclonal anti-IDO antibody (cat. no. sc-25809; 1:500 dilution), goat polyclonal anti-HO-1 antibody (cat. no. sc-1796; 1:500 dilution) and goat polyclonal anti-IL-7 antibody (cat. no. sc-1268; 1:500 dilution) at 4°C overnight or 37°C for 2 h. Subsequently, the membranes were incubated with peroxidase-conjugated goat anti-rabbit immunoglobulin (Ig)G and rabbit anti-goat IgG secondary antibodies (cat. no. BA-5000 1:20,000 dilution) at 37°C for 1 h. The protein-antibody complexes were visualized using an Enhanced Chemiluminescence (ECL) Detection system (Pierce ECL Western Blotting Substrate; Thermo Fisher Scientific). The integrated optical density (IOD) values for the target proteins and β-actin (internal reference; cat. no. bs-0061R; 1:1,500; Bioss Inc., Woburn, MA, USA) were obtained using the Quantity One (4.4.0) software (Bio-Rad, Hercules, CA, USA). The IOD_sample_/IOD_β-actin_ ratio reflects the relative content of each target protein. Larger IOD_sample_/IOD_β-actin_ ratios indicate higher relative target protein content.

### Cell immunocytochemistry

The HBZY-1 cells were incubated with various immunosuppressants for 6, 12 or 24 h. Following treatment with the various immunosuppressants, the cells were fixed with 4% paraformaldehyde (Wuhan Boster Biological Technology Co., Ltd., Wuhan, China) at room temperature for 15 min and then stored at 4°C. The immunocytochemistry kits used for detection of IDO, HO-1 and IL-7 included Envision™ (anti-rabbit IgG stock solution; cat. no. K500711; Dako) and PV-9003 (anti-goat IgG stock solution; Beijing Zhongshan Golden Bridge Biological Technology Co., Ltd.). Endogenous peroxidase activity was blocked with 3% hydrogen peroxide at room temperature for 10 min, then the samples were washed in PBS 3 times, each for 2 min. They were then incubated with the primary antibodies at 4°C overnight. The primary antibodies used in the immunocytochemical assay, for the detection of IDO, HO-1 and IL-7 were the same as those used in the western blot analysis, albeit at a 1:50 dilution. The samples were then incubated with the Envision rabbit and rat universal secondary antibody at 37°C for 45 min, then PV-9003 kit reagent 1 (100 *µ*l) was added at 37°C for 20 min, then the samples were subjected to 3 washes with PBS. The PV-9003 kit reagent 2 (100 *µ*l) was then added at 37°C for 20 min, then the samples were washed in PBS. The samples were then dehydrated with gradient alcohol, then permeabilized in xylene, prior to mounting with neutral gum. Samples treated with PBS instead of the primary antibody were used as a negative control. The target proteins were stained with 3,3′-diaminobenzidine chromogenic reagent (Thermo Fisher Scientific). The cells positive for target gene expression exhibited clear brown-yellow staining. Images of the cells were captured using an Olympus microscope (BX51; Olympus Corporation, Tokyo, Japan) and were processed using Adobe Photoshop version 7.0 software (Adobe Systems, San Jose, CA, USA).

### Statistical analysis

Values are presented as the mean ± standard deviation. Significant differences between the groups were analyzed using univariate analysis of variance. P<0.05 was considered to indicate a statistically significant difference. Statistical analyses were performed using SPSS version 14.0 software (SPSS Inc., Chicago, IL, USA).

## Results

### Effects of immunosuppressive drugs on the mRNA expression levels of IDO, HO-1 and IL-7 in mesangial cells, as detected by RT-qPCR

**Figure 1:**
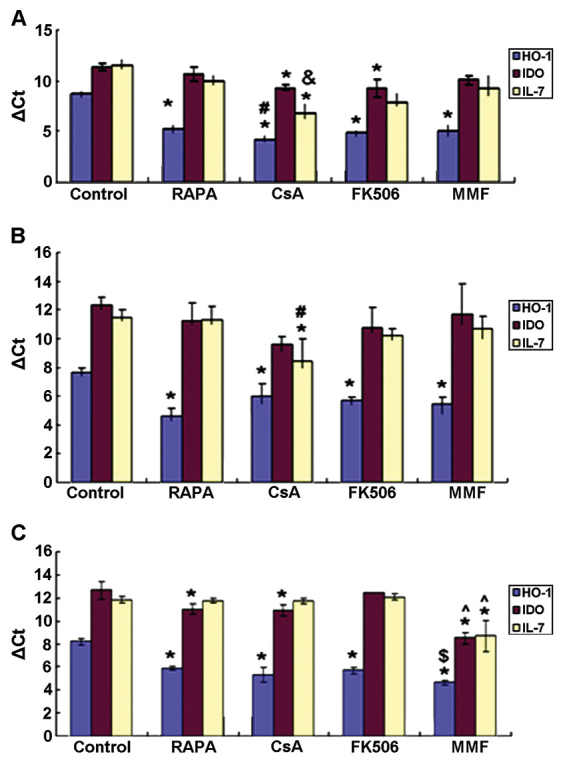
mRNA expression levels of HO-1, IDO and IL-7 in the HBZY-1 mesangial cells (A) 6 h; (B) 12 h; and (C) 24 h after administration of the immunosuppressive agents. ^*^P<0.05 vs. control group, ^#^P<0.05 vs. RAPA group, ^&^P<0.05 vs. RAPA and MMF groups, ^^^P<0.05 vs. other experimental groups, ^$^P<0.05 vs. RAPA and Tac groups. IDO, indoleamine 2,3-dioxygenase; HO-1, heme oxygenase-1; IL-7, interleukin-7; RAPA, rapamycin; CsA, cyclosporine A; FK506, tacrolimus; MMF, mycophenolate mofetil; ΔCt, Δ cycle threshold.

As shown in Fig. 1A, at 6 h, the mRNA expression levels of IDO were markedly elevated in the CsA and Tac groups as compared with those in the control group. Furthermore, the mRNA expression levels of HO-1 were significantly lower in the control group as compared with those in the other experimental groups (P<0.05). The mRNA expression levels of HO-1 were higher in the CsA group as compared with those in the RAPA group. No significant differences were detected in the HO-1 mRNA expression levels between the CsA, Tac and MMF groups. The mRNA expression levels of IL-7 were significantly higher in the CsA group as compared with those in the RAPA, MMF and control groups (P<0.05).

As shown in Fig. 1B, after 12 h, no significant differences were observed in the mRNA expression levels of IDO between the various experimental groups. The mRNA expression levels of HO-1 were significantly lower in the control group as compared with those in the drug treatment groups (P<0.05). However, no significant differences were detected in the HO-1 mRNA expression levels among the various drug treatment groups. The mRNA expression levels of IL-7 were significantly increased in the CsA group as compared with those in the RAPA and control groups (P<0.05).

As shown in Fig. 1C, after 24 h, the mRNA expression levels of IDO were significantly higher in the MMF group as compared with those in the other experimental groups (P<0.05). In addition, the mRNA expression levels of IDO were markedly increased in the CsA and RAPA groups as compared with those in the control group (P<0.05). The mRNA expression levels of HO-1 were significantly lower in the control group as compared with those in the other groups (P<0.05), and were markedly higher in the MMF group as compared with those in the RAPA and Tac groups (P<0.05). Furthermore, no differences were observed in the mRNA expression levels of HO-1 between the CsA and Tac groups. The mRNA expression levels of IL-7 were significantly elevated in the MMF group as compared with those in the other groups (P<0.05).

### Expression of IDO, HO-1 and IL-7, as detected by cell immunohistochemistry

**Figure 2:**
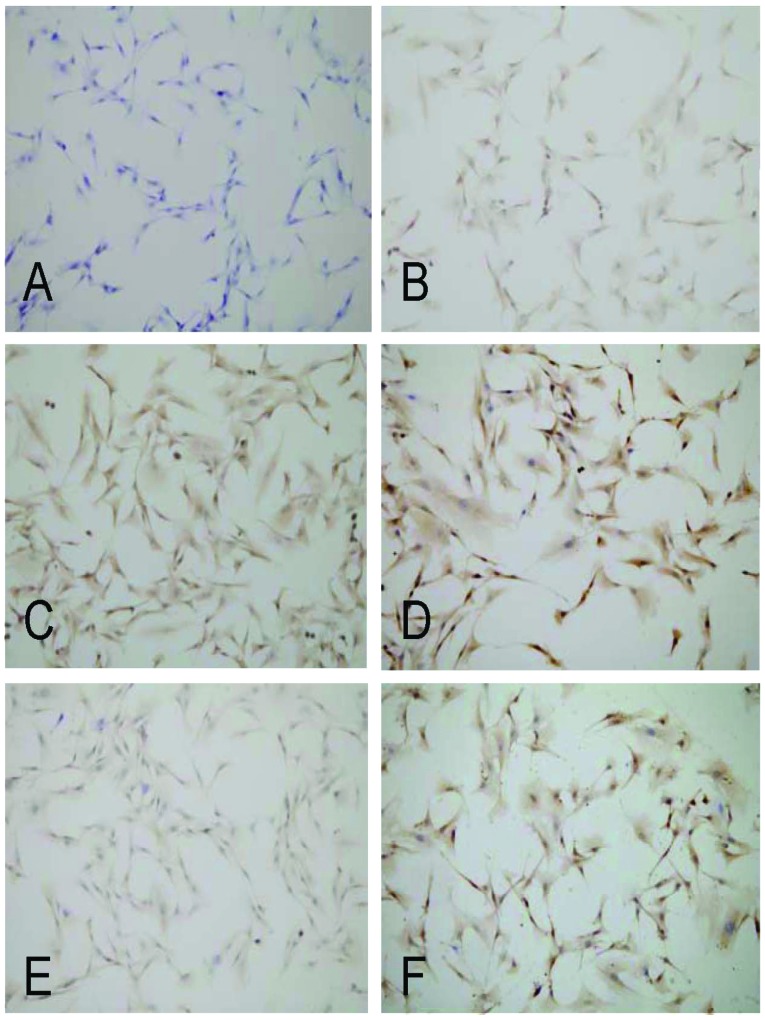
Effects of the various immunosuppressive agents on the expression of IDO in HBZY-1 mesangial cells at 24 h (immunocytochemistry; magnification, x80). IDO was markedly expressed in the caryon and cytoplasm of cells treated with MMF, CsA and Tac, particularly in those treated with MMF. (A) negative control; (B) control; (C) CsA group; (D) MMF group; (E) RAPA group; and (F) Tac group. IDO, indoleamine 2,3-dioxygenase; RAPA, rapamycin; CsA, cyclosporine A; Tac, tacrolimus; MMF, mycophenolate mofetil.

**Figure 3:**
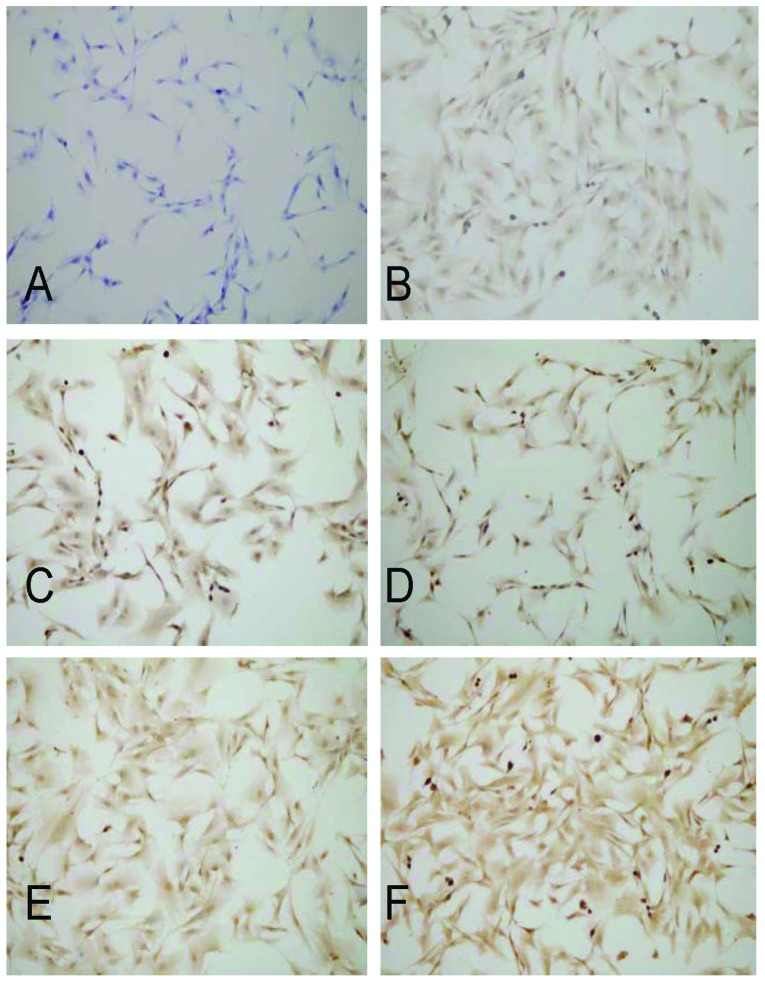
Effects of the various immunosuppressive agents on the expression of HO-1 in HBZY-1 mesangial cells at 24 h (immunocytochemistry; magnification, x80). HO-1 was intensively expressed in the caryon and cytoplasm in MMF, CsA and Tac groups, particularly in the MMF group.(A) negative control; (B) control; (C) CsA group; (D) MMF group; (E) RAPA group; (F) Tac group. HO-1, heme oxygenase-1; RAPA, rapamycin; CsA, cyclosporine A; Tac, tacrolimus; MMF, mycophenolate mofetil.

**Figure 4:**
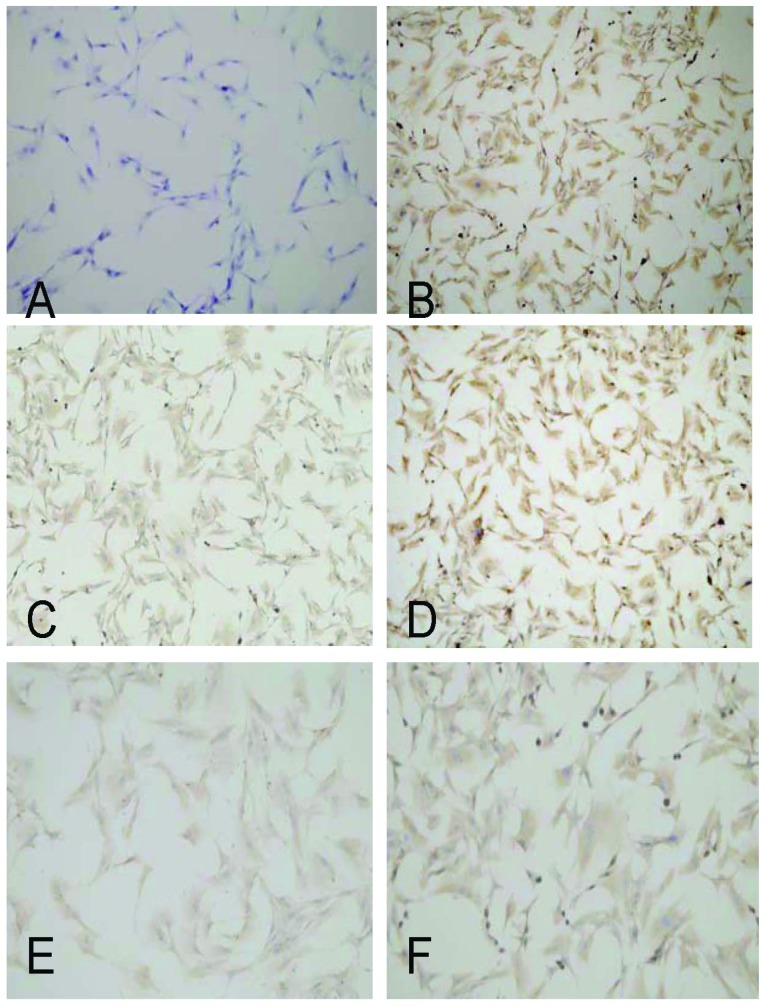
Effects of the various immunosuppressive agents on the expression of IL-7 in HBZY-1 mesangial cells at 24 h (immunocytochemistry; magnification, ×80). IL-7 was intensively expressed in the caryon and cytoplasm in MMF, CsA and Tac groups, particularly in the MMF group. (A) negative control; (B) control; (C) CsA group; (D) MMF group; (E) RAPA group; (F) Tac group. IL-6, interleukin-7; RAPA, rapamycin; CsA, cyclosporine A; Tac, tacrolimus; MMF, mycophenolate mofetil.

As shown in Fig. 2, IDO was markedly expressed in the caryon and cytoplasm of the mesangial cells treated with MMF, CsA and Tac, particularly in those treated with MMF. As shown in Fig. 3, HO-1 was markedly expressed in the caryon and cytoplasm of all of the treatment groups at 24 h; however, there were no significant differences between them. As shown in Fig. 4, IL-7 was predominantly expressed in the cytoplasm of the mesangial cells, and was most highly expressed in the cells treated with MMF at 24 h.

### Protein expression levels of IDO, HO-1 and IL-7

**Figure 5:**
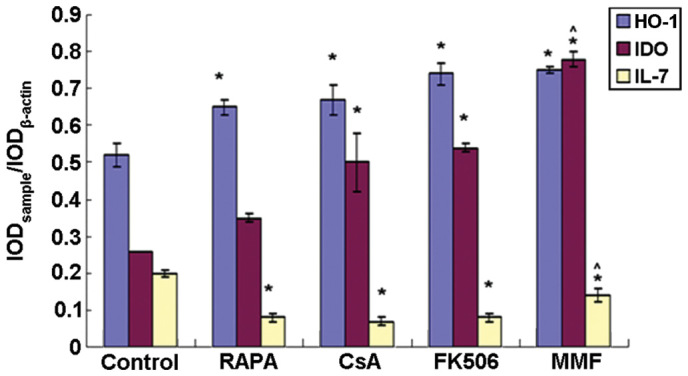
Protein expression levels of HO-1, IDO, and IL-7 at 24 h after the administration of various immunosuppressive agents. ^*^P<0.05 vs. control group, ^^^P<0.05 vs. other experimental groups. IDO, indoleamine 2,3-dioxygenase; HO-1, heme oxygenase-1; IL-7, interleukin-7; RAPA, rapamycin; CsA, cyclosporine A; FK506, tacrolimus; MMF, mycophenolate mofetil; IOD, integrated optical density.

At 24 h, the protein expression levels of HO-1 were markedly elevated in the CsA, Tac, RAPA and MMF groups, as compared with those in the control group (P<0.05). Furthermore, no significant differences were detected in the protein expression levels of HO-1 between the drug treatment groups. The protein expression levels of IDO were significantly increased in the CsA, Tac and MMF groups as compared with those in the control group (P<0.05). The protein expression levels of IDO and IL-7 were significantly higher in the MMF group as compared with those in the CsA, Tac and RAPA groups (P<0.05). There were no significant differences observed between the Tac and CsA groups. In addition, RAPA had no significant effect on the protein expression levels of IDO. The protein expression levels of IL-7 were markedly decreased in the CsA, Tac, RAPA and MMF groups as compared with those in the control group (P<0.05). No significant differences were detected in the protein expression levels of IL-7 between the RAPA, CsA and Tac groups (Fig. 5).

## Discussion

IDO is a potent immunosuppressive enzyme that has gained attention in recent years. IDO is a 45-kDa monomeric heme-containing protein and the rate-limiting enzyme of the kynurenine pathway, which degrades the essential amino acid tryptophan (9–11). IDO-induced tryptophan degradation inhibits the proliferation of T cells, accelerates T-cell apoptosis and triggers cell cycle arrest (12,13,25). In addition, the overexpression of IDO has been shown to reduce the incidence of graft injury or rejection through inhibiting alloreactive T-cell responses and enhancing the local antioxidative defense system, leading to peripheral tolerance and long-term graft survival (12,13). However, inhibition of IDO activity in graft-tolerant rats has been shown to lead to rapid graft rejection (26).

HO is the rate-limiting enzyme in the conversion of ferroheme to billiverdin, free iron ions and carbon monoxide (14–20). To date, three HO isoforms have been identified. HO-2 is the constitutively expressed isoform, whereas HO-1 is the inducible isoform, which is typically expressed at a rather low level in the majority of cell types. The expression of HO-1 may be induced by various stimuli, such as ferroheme, heavy metals, hyperoxia, hypoxia, tissue plasminogen activator, cytokines and chemokines (21–23,27,28). The third isoform, HO-3, has yet to be fully defined (21–23,27,28). HO-1 and its metabolites represent a cytoprotective defense mechanism in various models of cellular and tissue damage and exhibit anti-inflammatory, anti-apoptotic, anti-oxidative, anti-proliferative, anticoagulant and vasodilatory effects through various pathways (21,29–31). Previous studies have demonstrated that HO-1 has a protective role in numerous organ transplantation models (29–33). HO-1 has also been shown to reduce leukocyte-endothelial interactions following ischemia/reperfusion (I/R) injury, alleviate chronic rejection and improve graft survival (29,32,33). Furthermore, deficient levels of HO-1 may lead to ferroheme aggregation in the circulation and vascular endothelial damage (21).

IL-7 is a potent survival factor for T cells, which has key roles in the maintenance of intracellular homeostasis and the regulation of the anti- and pro-apoptotic members of the B-cell lymphoma 2 protein family (34). IL-7 promotes the transition of CD4^+^ effector T cells to persistent memory T cells and is essential for the homeostasis, proliferation and survival of memory CD8^+^ T cells. It has previously been suggested that targeting IL-7 during transplantation may inhibit the production of allogeneic antigen-specific memory T cells, thus promoting the induction of graft tolerance (12).

CAN is a major limiting factor of long-term graft survival following kidney transplantation (6), which may be induced by various immune and non-immune factors, including I/R injury and treatment with immunosuppressants. The immunosuppressive drugs CsA and Tac have been identified to promote the incidence of CAN (35). The expression levels of IDO, HO-1 and IL-7 in renal grafts, and the effects of various immunosuppressants on the expression of these genes, are yet to be fully elucidated. Therefore, the present study aimed to improve the understanding of the mechanisms of action of immunosuppressants following transplantation.

In the present study, a model of glomerular mesangial cell injury was established and investigated. Treatment with CsA and particularly with MMF significantly upregulated the mRNA and protein expression levels of IDO. FK-506 (Tac) also markedly upregulated the protein expression levels of IDO. Conversely, RAPA exhibited no apparent effect on IDO protein expression. These results indicated that MMF, and the calcineurin inhibitors (CNIs) Tac and Csa may have an important role in the regulation of IDO expression. In addition, RAPA and FK-506 were shown to regulate IDO expression at the transcriptional and post-transcriptional level, respectively. This complementary association between RAPA and FK506 may provide a novel theoretical basis for the clinical application of a combination therapy of RAPA and FK-506.

MMF inhibits T-cell proliferation through blocking DNA synthesis, and CNIs, a class of T-cell directed immunosuppressive drugs, also suppress the activity of T-cells (36,37). The results of the present study suggested that MMF and CNIs may inhibit alloreactive T-cell responses through the tryptophan catabolic pathway following organ transplantation. This pathway may therefore account for another underlying mechanism of the prevention of graft rejection and CAN by MMF and CNIs.

Various drugs, including statins, RAPA, erythropoietin and probucol, enhance the expression of HO-1 (22). The results of the present study indicated that, in addition to RAPA, the immunosuppressants CsA, FK506 and MMF markedly upregulated the mRNA as well as protein expression levels of HO-1. No significant differences were observed in the HO-1 protein expression levels in the cells following treatment with CsA, FK506, RAPA or MMF, suggesting that CsA, FK506, RAPA and MMF exert similar inductive effects on HO-1 expression at the post-transcriptional level. The administration of CsA, FK506, RAPA and MMF following kidney transplantation is conducive to the prevention of I/R injury and the reduction of chronic rejection (33). In addition, these drugs have been shown to regulate fibrosis and angiogenesis (22). Therefore, the induction of HO-1 by CsA, FK506, RAPA and MMF is beneficial for the prevention and treatment of CAN.

Previous studies have demonstrated that IL-7-mediated homeostatic proliferation of T cells is regulated by MMF, but not by CNIs or sirolimus (38). The results of the present study demonstrated that although MMF significantly increased the mRNA expression levels of IL-7, protein expression levels of IL-7 were significantly inhibited by MMF and particularly by CNIs and RAPA. It is evident that all four types of immunosuppressants are involved in the regulation of IL-7 protein expression *in vitro*, suggesting that the four types of immunosuppressants regulate the proliferation of T cells. The present study also observed that MMF exerted bidirectional regulatory effects on the mRNA and protein expression levels of IL-7. Conversely, treatment with CNIs and RAPA exhibited no significant effect on the expression levels of IL-7 mRNA, but they did markedly inhibit the protein expression levels of IL-7. Inhibition of IL-7 protein expression by immunosuppressants may have an important role in the prevention of graft rejection. Clinical studies have demonstrated that prior to recovery, the plasma levels of IL-7 and CD8^+^ T cells are increased, suggesting that IL-7 may result in generation of transplant recipients’ CD8^+^ memory T cells (39,40); therefore, inhibition of the production of IL-7 may contribute to suppression of memory T cell production, and thus serve to prevent graft rejection and promote long-term allograft survival.

In conclusion, the present study investigated for the first time, to the best of our knowledge, the gene expression levels of IDO, HO-1 and IL-7 in glomerular mesangial cells *in vitro*, as well as the regulatory effects of various immunosuppressants on the expression of these genes. The expression levels of HO-1 were significantly upregulated in response to treatment with CsA, FK506, RAPA and MMF, whereas the expression levels of IL-7 were markedly downregulated by treatment with the above immunosuppressants. CsA, FK506 and MMF significantly enhanced the expression levels of IDO, whereas RAPA exhibited no apparent effect on IDO. To understand the effects of IDO, HO-1, and IL-7 expression on chronic graft rejection and CAN, further in-depth studies are required.

## Figures and Tables

**Table I:** Polymerase chain reaction primer and TaqMan^®^ probe sequences of each gene of interest.

Gene	Primer	Primer sequence	Amplification segment (bp)
IDO	F R TM	5′-TGGCAAACTGGAAGAAAAAG-3′ 5′-ATTGCTTTGGATTGCAGGAGAA-3′ 5′-TTTCCTGGTGGGGACTGCGA-3′	151
HO-1	F R TM	5′-GACAGCATGTCCCAGGATTT-3′ 5′-CATCACCAGCTTAAAGCCTT-3′ 5′-CACCTCCTTGGTGGCCTCCTTC-3′	135
IL-7	F R TM	5′-CATCAATCAACTGGACAAAATG-3′ 5′-GTCATTGAATTCCTCACTGAT-3′ 5′-CCTCAACTTGCGAGCAGCAC-3′	172
GAPDH	F R TM	5′-CCTCAAGATTGTCAGCAAT-3′ 5′-CCATCCACAGTCTTCTGAGT-3′ 5′-FAM-ACCACAGTCCATGCCATCAC-TAMRA-3′	141

F, forward primer; R, reverse primer; TM, TaqMan probe; IDO, indoleamine 2,3-dioxygenase; HO-1, heme oxygenase-1; IL-7, interleukin-7; bp, base pairs.
